# Factor XIII-A: An Indispensable “Factor” in Haemostasis and Wound Healing

**DOI:** 10.3390/ijms22063055

**Published:** 2021-03-17

**Authors:** Fahad S. M. Alshehri, Claire S. Whyte, Nicola J. Mutch

**Affiliations:** Aberdeen Cardiovascular & Diabetes Centre, School of Medicine, Medical Sciences and Nutrition, Institute of Medical Sciences, University of Aberdeen, Aberdeen AB25 2ZD, UK; r01fsma@abdn.ac.uk (F.S.M.A.); c.s.whyte@abdn.ac.uk (C.S.W.)

**Keywords:** Factor XIII-A, transglutaminase, cross-linking, cellular FXIII-A, haemostasis, wound healing

## Abstract

Factor XIII (FXIII) is a transglutaminase enzyme that catalyses the formation of ε-(γ-glutamyl)lysyl isopeptide bonds into protein substrates. The plasma form, FXIIIA_2_B_2_, has an established function in haemostasis, with fibrin being its principal substrate. A deficiency in FXIII manifests as a severe bleeding diathesis emphasising its crucial role in this pathway. The FXIII-A gene (F13A1) is expressed in cells of bone marrow and mesenchymal lineage. The cellular form, a homodimer of the A subunits denoted FXIII-A, was perceived to remain intracellular, due to the lack of a classical signal peptide for its release. It is now apparent that FXIII-A can be externalised from cells, by an as yet unknown mechanism. Thus, three pools of FXIII-A exist within the circulation: plasma where it circulates in complex with the inhibitory FXIII-B subunits, and the cellular form encased within platelets and monocytes/macrophages. The abundance of this transglutaminase in different forms and locations in the vasculature reflect the complex and crucial roles of this enzyme in physiological processes. Herein, we examine the significance of these pools of FXIII-A in different settings and the evidence to date to support their function in haemostasis and wound healing.

## 1. Background

**Figure 1:**
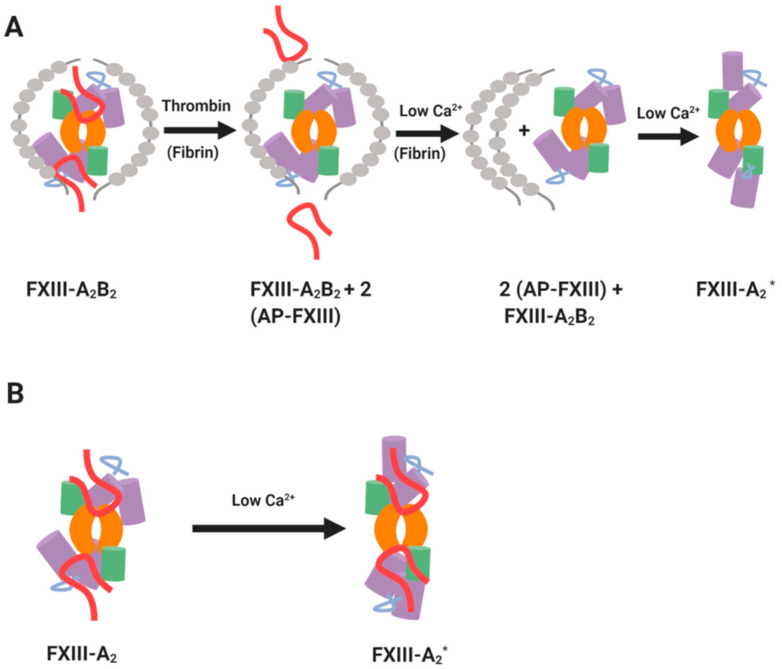
Mechanisms of FXIII activation. (**A**) Thrombin- and Ca^2+^-driven cleavage of FXIIIA_2_B_2_. (**B**) Non-proteolytic activation of cellular FXIII-A by low Ca²⁺. The green and purple cylinders represent β-barrel and β-sandwich domains of FXIII-A subunit, respectively. The core domains in FXIII-A subunit are shown in orange and the activation peptides are shown in red. The inhibitory B subunits are shown in grey. Adapted from the work in [1].

FXIII belongs to the transglutaminase family of enzymes which is named according to its crucial role in blood coagulation. FXIII is a zymogen that must be activated to reveal its transglutaminase function [1]. Plasma FXIII (pFXIII) circulates as a heterotetramer, termed FXIII-A_2_B_2_, which is comprised of two catalytic A subunits and two carrier B subunits which envelope the catalytic subunits [2,3]. pFXIII circulates at an average concentration of 68 nM [4]. The A subunits in plasma exist only in complex with the carrier B subunits, while an excess (43–62 nM) of B homodimers is evident in the circulation [4,5]. pFXIII is largely found in a non-covalent complex with one of its dominant substrates, fibrinogen (K_D_ = ~10 nM) [6]. Thrombin cleaves the Arg37-Gly38 peptide bond in the activation peptide (AP-FXIII) which flank the amino terminus of the A_2_ subunits thereby destabilising the complex (Figure 1A). Subsequent binding of Ca^2+^ promotes dissociation of the inhibitory FXIII-B_2_ subunits to release a functional transglutaminase enzyme, FXIII-A_2_*. Other serine proteases, such as the endogenous platelet acid protease [7] and calpain [8] can also reportedly activate FXIII. Fibrin acts a s a cofactor in activation of FXIII by forming a tertiary complex with thrombin and FXIII thereby promoting cleavage [6,9,10,11,12]. Once activated FXIII (FXIIIa) elicits transamidase activity that introduces ε-(γ-glutamyl)lysyl isopeptide cross-links into protein substrates. Crosslinks can form within a single substrate, such as fibrin, or between different proteins which can impact on their biological function [13].

The cellular form of FXIII is a homodimer of the A subunits, termed FXIII-A throughout this review [14]. Cellular FXIII-A is non-proteolytically activated to FXIII-A* by modest increases in intracellular Ca^2+^ concentrations (Figure 1B) [15,16]. FXIII-A exists in cells of bone marrow and mesenchymal lineage, notably platelets [17,18,19], megakaryocytes [20] monocytes [21,22], circulating [21,23] and tissue macrophages [23], dendritic cells [24], chondrocytes [25,26,27], osteoblasts [28] and preadipocytes [29]. Regulation of FXIII-A* is a complex area. It is clear that the regulatory “B” subunits function to attenuate FXIIIA_2_B_2_ in plasma and stabilise the complex in this milieu [30]. However, once activated, the story is more complex. Elegant studies have delineated a role for plasmin regulation of FXIIIa [31], but the FXIIIA_2_B_2_ complex is protected against degradation. There is also a suggested role of thrombin [32] and proteolytic enzymes secreted from granulocytes [33] in regulation of FXIII* function. The focus of this review will be on the localization and function of the FXIII-A subunit and examine its crucial function in regulation of haemostasis and wound healing.

## 2. Structural Considerations

**Figure 2:**
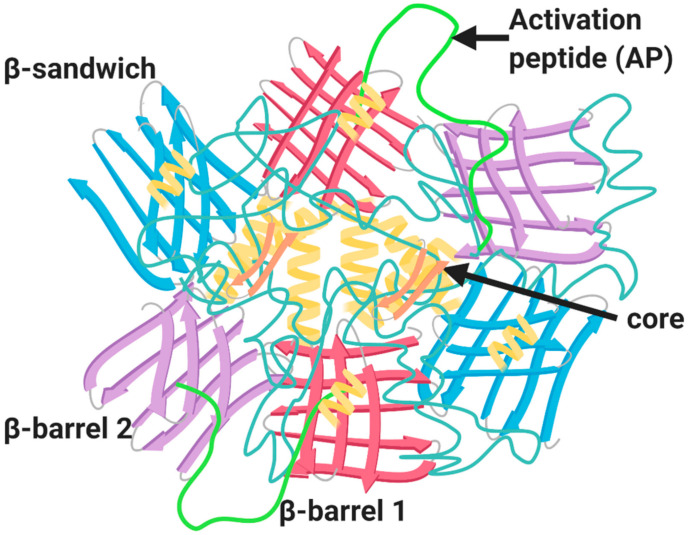
Structure of FXIII-A zymogenic homodimer. The structure of FXIII-A homodimer is composed of four major domains: catalytic core domain (orange), β-sandwich domain (blue), β-barrel-1 domain (red) and the β-barrel-2 domain (purple); alpha helices strands (pale yellow); and the activation peptide (AP) (green).

Identification and cloning of the F13A1 and F13B genes led to recombinant expression of the FXIIIA_2_ and FXIIIB_2_ subunits [34]. This led to a description of the zymogenic form of the A_2_ homodimer [35,36] and subsequently a Ca^2+^ activated and inhibitor stabilized FXIIII-A subunit [37]. The structure of the plasma and cellular forms is identical, which provided an early clue that the plasma pool of FXIII-A was of haematopoietic origin. FXIII-A (Figure 2; 83 kDa) is comprised of several domains including the activation peptide (AP-FXIII (1–37), β-sandwich domain (38–184), the catalytic core domain (185–515), β-barrel-1 domain (516–628) and β-barrel-2 domain (629–731) [38]. The catalytic core domain is largely comprised of helical structures; however, the rest of the domains contain β-sheets with limited helical elements [38] (Figure 2). The catalytic cores of two FXIII-A subunits align to form the FXIII-A_2_ dimer which is encased by the six β-sheet domains [39]. The active site cysteine residue (Cys314) is completely encased by the AP-FXIII, to impede interaction with target substrates [35]. Dissociation of AP-FXIII promotes structural rearrangement of the catalytic triad (Cys314; His373; Asp396) to allow docking of substrate to the active site. There is also a single Ca^2+^ binding site per FXIII-A subunit that is crucial for activation of the transglutaminase [40,41].

FXIIIa uses a double displacement mechanism for cross-linking proteins. In the initial stage, Cys314 attacks the glutamine γ-carboxyamide group of a glutamine acceptor protein, displacing an ammonia molecule to form a thioester intermediate. In the second stage, the reactive thioester intermediate is attacked by the lysine ϵ-amino group of the amine donor protein, thereby displacing Cys314 and generating an isopeptide bond between the two substrate proteins and the concomitant release of FXIIIa [42]. In the absence of lysine residues, water reacts with the thioester intermediate converting glutamine into glutamic acid [42]. Glutamine sites that participate in FXIIIa-catalysed reactions have been identified through incorporation of primary amines, such as dansylcadaverine [43,44,45,46,47] and 5-(biotinamido)pentylamine [48,49]. Alternatively, labelled synthetic peptides have been designed to incorporate into the lysine residues of FXIIIa substrates [44]. In several cases, reactive glutamine and lysine residues have been characterised by mass spectrometry and Edman sequencing analysis [48,49,50].

## 3. Pools of FXIII-A within the Vasculature

The presence of the FXIII-A subunit in plasma has posed a conundrum: (i) it is not released from hepatocytes alongside its regulatory subunit FXIII-B, (ii) the concentration of FXIII-A is relatively high within the circulation and (iii) it lacks an identifiable endoplasmic reticulum signal sequence beseeching the question of how it can be released within the plasma environment. The most abundant source of FXIII-A is within the cytoplasm of cells of bone marrow and mesenchymal lineage. Bone marrow transplantation studies in humans implicated platelets, macrophages and unidentified circulatory haematopoietic cells as the source of plasma FXIII-A [51,52,53,54]. Cordell et al. [55] demonstrated that platelets were not the source of plasma FXIII-A, as thrombocytopenic mice exhibit normal levels of plasma FXIII-A [55]. Elegant studies from the same group used a complex series of lineage specific *Cre* mice to demonstrate that resident macrophages maintain plasma FXIII-A and excluded the megakaryocytic lineage as a major contributor. Externalisation of the closely related family member transglutaminase 2 has been shown on the surface of macrophages, but FXIII-A was not evident [56]. The enigma of how FXIII-A is released from these cells remains; nonetheless, current evidence underscores the importance of tissue specific macrophages in the release of this key protein to the blood stream.

### 3.1. Platelet-Derived FXIII-A

Platelet FXIII-A is synthesised in the precursor megakaryocyte cell during thrombopoiesis and packed during pro-platelet production. Unlike the majority of platelet-derived coagulation factors, FXIII-A is not deposited within the α-granules but is instead found in cytoplasm, most likely due to the lack of ER signal to direct it to the granule cargo. FXIII-A is an abundant protein within the cytoplasm, with levels as high as 60 ± 10 fg/platelet, accounting for approximately 3% of total protein [57]. Platelets therefore harbour approximately150-fold higher concentrations of FXIII-A than plasma, thereby insinuating this pool may be important in certain physiological functions [58]. Importantly, the Muszbek laboratory revealed that platelet FXIII-A can be activated within the cytoplasm following elevation of intracellular Ca^2+^ during platelet activation [16]. This thrombin-independent process occurs without concomitant release of the activation peptide [16] (Figure 1). Endocytosis of pFXIII into platelet α-granules during their circulation has been reported [59,60,61], but negligible amounts are detectable within the platelet releasate [62,63,64]. In line with these observations, FXIII-A levels are normal in platelets derived from patients with Grey Platelet Syndrome [65] and levels are unchanged in thrombocytopenic mice [55].

**Figure 3:**
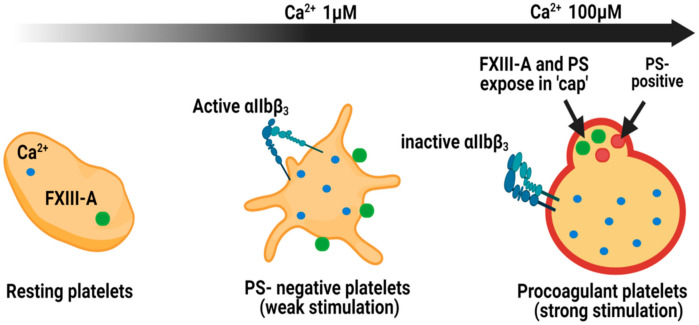
Localisation of FXIII-A in platelet subpopulations. Within resting platelets, Ca^2+^ ions and cellular FXIII-A reside within the cytoplasm. Following weak stimulation, with agonists such as ADP, PS-negative platelets expose FXIII-A on the external membrane. The integrin αIIbβ3 is exposed in this subpopulation of platelets. Stimulation with dual agonists, such as thrombin and collagen, generate procoagulant platelets which expose FXIII-A within the phosphatidylserine (PS)-rich cap. In this subpopulation αIIbβ3 is inactive [70]. The cap is also rich in other coagulation factors, such as factor X, factor IX and prothrombin, and substrates for FXIII-A including fibrinogen, thrombospondin and factor Va [64].

The absence of FXIII-A in the secretome of platelets led to the assumption that this pool did not contribute to haemostasis [66]. Our laboratory has revealed that FXIII-A is translocated from the cytoplasm to the outer leaflet of the membrane in stimulated platelets [64]. This pool of FXIII-A, while adhered to the membrane, is functional in conferring resistance against fibrinolytic degradation via the cross-linking of α_2-_antiplamin (α_2_AP) to fibrin [64]. The localisation of FXIII-A on the surface of phosphatidylserine-positive (PS-positive) platelets or procoagulant platelets occurs in the platelet “cap” or platelet body (Figure 3) [64]. These procoagulant platelets, as their name indicates, support assembly of coagulation factors which promote thrombin generation and subsequent fibrin formation [67]. Indeed, many other coagulation factors including factor Va, factor VIII, factor IXa, factor X/Xa, and prothrombin are concentrated within the “cap” region alongside FXIII-A [68]. The primary substrate of FXIII-A, fibrin(ogen), and other substrates including thrombospondin [69,70], and factor Va [43] are also abundant within the “cap” region of procoagulant platelets [71,72]. It has been suggested that fibrin and the integrin αIIbβ_3_ are critical for FXIII-A binding to the “cap” [68,73]. This localises FXIII-A in a prime location in which to promote crosslinking of fibrin and substrates into the fibrin matrix. However, αIIbβ_3_ is proposed to be inactive on procoagulant platelets, due to high Ca^2+^ and calpain levels [74]_._ The “cap” region of procoagulant platelets has been proposed as a point of attachment to aggregates and the forming clot [68]. Cross-linking of adhesive proteins, fibrin and thrombospondin in the “cap” could be a vital element in consolidating the attachment of procoagulant platelets under shear stress.

PS-negative, spread platelets, also expose FXIII-A where it is diffusely localised across the membrane (Figure 3) [64]. The discrete role played by these platelets in thrombus initiation, propagation and in particular the expression of the active integrin αIIbβ_3_ and binding of fibrinogen most likely account for this differential staining pattern of FXIII-A. Of interest to consider is the difference in intracellular Ca^2+^ spikes associated with these platelet subpopulations following stimulation. Procoagulant platelets undergo a massive increase in cytosolic Ca^2+^ [68,75,76], with a recent report suggesting it is around 100-fold higher than spread, PS-negative platelets [77]. It is therefore feasible that the activity of FXIII-A on the surface of different subpopulations of platelets is hugely different and requires further investigation.

Clot retraction is platelet-mediated process that serves to constrain clot volume [78,79], thereby reducing blood loss [80] and permitting blood flow past otherwise obstructive thrombi [81]. The integrin αIIbβ3 acts as a molecular bridge between extracellular fibrinogen and the intracellular actin cytoskeleton via sphingomyelin-rich lipid rafts [82]. The cytoskeleton interacts with the α3 subunit tails via the adapter proteins talin and vinculin [83]. During clot retraction, fibrin bound to αIIbβ3 triggers outside-in signalling [84], resulting in the contraction of the actin cytoskeleton. FXIIIA_2_B_2_ contributes to the strength and rigidity of the condensed clot by cross-linking fibrin, and enhancing platelet spreading [85]. Conflicting evidence exists on the effects of platelet FXIII-A on clot retraction. Early reports found that clot retraction was normal in FXIII-deficient patients [86,87,88]. However, Kasahara et al. [82,89] demonstrated that clot retraction was significantly impaired in the absence of platelet FXIII-A transglutaminase activity in platelet-rich plasma from FXIII-A knockout mice [82,89]. Nevertheless, platelet FXIII-A has been shown not to contribute to retention of red blood cells [90]. It is apparent that further studies are required into the role of platelet FXIII-A in clot retraction in vivo. In procoagulant platelets, FXIII-A and calpain act in concert to downregulate integrin αIIbβ3 adhesive function, thereby limiting platelet recruitment in to the forming aggregate [91]. This FXIII-A-dependent mechanism attenuates thrombus size and may be important in preventing haemostatic clots from becoming obstructive in the vasculature.

### 3.2. Monocyte/Macrophage-Derived FXIII-A

FXIII-A is expressed on the cell surface of monocytes and macrophages [92] in response to stimulation with certain immune modulators, which is akin to the situation in platelets [64]. Indeed, monocytes isolated from patients with a congenital deficiency in F13A1 show a lack of FXIII-A and transglutaminase activity [93]. Within monocytes/macrophages, FXIII-A is localised to the cytoplasm but several studies have indicated it can be shuttled to the surface [94,95,96], and it is secreted by dendritic cells into the culture medium [97]. Secretion by an alternative secretory pathway has been proposed given the lack of ER signal in the FXIII-A protein [98]. Translocation of FXIII-A to the nucleus of differentiating human macrophages has been reported but the exact function of this transglutaminase enzyme in this locale is unclear [99].

The expression of FXIII-A in macrophages is dynamic in nature and is modulated in response to external stimuli and the phenotype of the activated macrophage. Macrophages can be “alternatively” or “classically” activated depending on the activating stimulus. “Classically activated”, or M1, macrophages are generated in response to stimulation with the immune mediators, interferon-γ, lipopolysaccharide or tumour necrosis factor [100]. These proinflammatory “type 1” macrophages [101] tend to exhibit down-regulation of FXIII-A [102,103]. “Alternatively activated”, or M2 macrophages, are stimulated in response to anti-inflammatory mediators, such as interleukin-4 and -13 [101]. M2 macrophages are reported to function in matrix remodelling, wound healing, allergy and parasite killing [100] and it is this subtype of macrophages that reveal upregulation of FXIII-A [103,104,105]. The selective expression of FXIII-A in M2 macrophages is in line with the capacity of this transglutaminase to act as an anti-inflammatory and pro-wound healing molecule.

Phagocytosis is the active ingestion and breakdown of microbes or other foreign particles by cells such as monocytes and macrophages. Phagocytic processes are driven by a finely controlled rearrangement of the actin cytoskeleton [106]. Considering the key role of FXIII-A in regulating cytoskeletal proteins, it is perhaps not surprising that it is directly linked to this process [107,108,109]. Studies have indicated that FXIII-A activity may play a role in increasing phagocytosis in monocytes and macrophages [110]. Phagocytosis is positively correlated with FXIII-A expression in myelomonocytic cells [111]. In accordance with this, Fcγ and complement receptor-mediated phagocytosis is impaired in monocytes and macrophages following inhibition of FXIII-A in FXIII-A-deficient mice [110]. Similarly, phagocytosis is significantly attenuated in monocytes isolated from FXIII-A deficient patients [110]. FXIII-A is upregulated during monocyte-derived dendritic cell differentiation and supports migration of mature cells [112]. The role of monocyte/macrophage FXIII-A in haemostasis has not been widely explored; however, these cells can promote cross-linking of fibrin [92,95], suggesting a potential role in thrombus stabilisation. Interestingly, thrombin treatment of monocytes does not augment exposure of FXIII-A [92], suggesting these cells may contribute to haemostasis in a situation where there is also an increase in the type 2 immune response, for example, in a wound healing capacity. Together, these data implicate FXIII-A in the phagocytic and/or migration capacity of these cells suggesting an important function of this pool of FXIII-A in innate immunity, inflammation and wound healing. However, there are many unaddressed questions in relation to the externalisation of FXIII-A on these cells. The dominant bleeding phenotype in congenital FXIII-A deficiency has perhaps masked the auxiliary roles of this transglutaminase in innate immunity, inflammation and wound healing. Nonetheless, it is apparent that there is an association with depleted levels of circulatory FXIII-A and delayed wound healing in different settings, such as venous leg ulcers, and in chronic inflammatory conditions, including inflammatory bowel disease [113,114,115].

## 4. FXIII Deficiency and Associated Complications

### 4.1. Congenital Deficiency

Congenital FXIII deficiency is a rare haemorrhagic disorder with an estimated prevalence of one per two million [116]. Umbilical stump bleeding in infants within the first few days of life is emblematic and frequently leads to a positive diagnosis. Patients also exhibit soft tissue haematoma, recurrent miscarriage and prolonged wound healing and most acutely intracranial bleeding [3]. Diagnosis of FXIII deficiency is challenging as routine coagulation tests, such as prothrombin time (PT), activated partial thromboplastin time (APTT), thrombin time (TT) and platelet count, are normal [117]. Laboratory tests employed to diagnose FXIII deficiency include clot solubility assays; quantitative FXIIIa activity assays; ELISA for the A and B subunits and A_2_B_2_ complex; and genetic testing [117,118,119]. More recently, a whole blood clot retraction assay has been reported to be valuable in diagnosis of FXIII deficiency and monitoring of treatment [120]. The International Society on Thrombosis and Haemostasis Scientific and Standardization Committee on Fibrinogen and Factor XIII recommended an algorithm to guide laboratory diagnosis of FXIII deficiency and classify the form of the disease [121]. Challenges remain in that many of these specialised tests are not universally available.

FXIII deficiency is classified into three groups: type I FXIII-A subunit deficiency, type II FXIII-A subunit deficiency and FXIII-B subunit deficiency [118]. Type I is a quantitative defect resulting from decreased synthesis of the protein, whereas type II deficiency is associated with normal levels of FXIII that is functionally deficient. FXIII-B subunit deficiency is exceptionally rare and is associated with a milder bleeding phenotype [3]. The milder penetrance of B subunit deficiency is perhaps not surprising given the fact that the active A subunits still circulate, albeit at a reduced half-life, due to the absence of the inhibitor subunits [122]. Over 70 causative mutations have been described in the FXIII-A gene, while only five have been reported in the FXIII-B gene [118].

Congenital FXIII-A deficiency is associated with spontaneous miscarriage within the first trimester [123]. Similarly, homozygous FXIII-A knockout mice die due to massive uterine bleeding events at approximately 10 days of gestation [124]. FXIII-A is located in the histocytes of the uterus [125], in tissue macrophages and in the placenta [126], and enhances the formation of cytotrophoblastic shell and the cross-linking of Nitabuch’s fibrinoid layers [127]. It is found to directly co-localise with fibrinogen and fibronectin at the Nichbuch layer and is likely to have a protective function by downregulating fibrinolysis within this region. Insufficient development of the cytotrophoblastic shell and Nitabuch’s fibrinoid layers leads to placental abruption and foetal loss [128]. Macrophages manifest in the Nitabuch’s layer and surrounding implantation tissue, suggesting FXIII-A in this region is of cellular origin. Nevertheless, supplementation of plasma with FXIII-A concentrate to trough levels of around 10% is sufficient to permit successful full-term pregnancy [3,123,129]. This poses a conundrum as to the origin of FXIII-A in the placental tissue considering the FXIII-B subunit is absent.

### 4.2. Pregnancy Complications

During normal pregnancy, levels of many coagulation factors, including fibrinogen and coagulation factors VII, VIII, IX and X, steadily increase [130]. Interestingly, the same is true of the FXIII-B subunit while the levels of FXIII-A, and accordingly the tetrameric complex FXIIIA_2_B_2_, decrease to approximately 50% during gestation [131,132,133]. Recurrent pregnancy loss is only associated with deficiencies in fibrinogen or FXIII, reflecting the key contribution of these coagulation factors in stabilization of the placenta [116]. There are limited studies analysing the perturbation of FXIII-A in recurrent pregnancy loss with individuals that are otherwise competent for FXIII-A [134,135]. Neither of these studies were able to establish a direct link between consecutive miscarriage and reduced levels of FXIII-A. It is feasible that mild depletion of FXIII-A in plasma is not sufficient to induce complications in placental development, or that other circulatory pools of FXIII-A can compensate. Indeed, the source of FXIII-A in the placenta is unclear. Clearly there is many unanswered questions pertaining to the role and origin of FXIII-A in pregnancy and whether disruptions to these circulatory pools could explain recurrent spontaneous miscarriage in individuals who do not harbour a FXIII-A deficiency.

### 4.3. Acquired FXIII Deficiency

Acquired FXIII deficiency is a rare condition in which circulating levels of FXIII can drop to 20–70% of normal due to decreased synthesis or increased consumption of FXIII in different disease states [136]. This can be due to conditions such as disseminated intravascular coagulation (DIC), sepsis, pulmonary embolism, stroke, liver cirrhosis, Crohn’s disease and ulcerative colitis or due to major surgery and trauma [121]. Alternatively, autoantibodies which prevent activation of FXIII, impair binding of FXIII to its substrate and cofactor fibrin, or attenuate the half-life of FXIII in plasma may develop [137]. Several cases relate to patients with systemic lupus erythematosus [138]. The most common clinical feature of acquired FXIII deficiency is haemorrhage in soft tissue. Decreased synthesis of FXIII can be observed in patients undergoing chemotherapy [53] and with chronic liver failure [139]. In a case of acquired FXIII deficiency, decreased thrombus stability was associated with defective crosslinking of α_2_AP [64].

## 5. The Indispensable Role of FXIII-A Haemostasis and Wound Healing

Proteomic approaches have identified over 147 potential substrates for FXIIIa in plasma [140]. The identified substrates are involved in processes such as haemostasis, complement, extracellular matrix organisation and inflammatory and immune response thereby illustrating the diverse and crucial function of FXIII-A in normal physiology. Of these substrates 48 were cross-linked into the forming fibrin matrix during formation thereby localising them at the site of injury and subsequent wound healing. Historically, it was viewed that as little as 5% FXIII is sufficient for crosslinking function of FXIII; however, this assumption was largely based on coagulation assays. Our own work on the antifibrinolytic function of FXIII reveal that replenishment to around 50% of circulating levels is necessary for normal haemostasis [141,142].

### 5.1. FXIII in Haemostasis

The role of plasma FXIII-A in haemostasis is well-established; it confers mechanical stability to thrombi by cross-linking the α- and γ-chains of fibrin, and provides protection against fibrinolytic breakdown by cross-linking fibrinolytic inhibitors to fibrin [44,45,143]. Cross-linking of fibrin reduces the association rate of plasmin for fibrin more than 6-fold [144]. In addition, multiple lysine residues within the C-terminal domain of fibrin act as a substrate for FXIIIa [145]. Cross-linking within these areas has the potential to mitigate binding of plasminogen and tPA to the fibrin network, thereby downregulating fibrinolysis. Another proposed mechanism by which FXIIIa confers resistance to fibrinolytic degradation is by compacting the fibrin fibre diameter and increasing fibre density in the clot [146]. Clots comprised of thinner fibres with smaller pores have previously been shown to show enhanced resistance to fibrinolysis [147], which can be ascribed to reduced solute access and to a reduction in binding of tPA [141,148,149].

Our laboratory illustrated that flow or shear stress is necessary to visualize the impact of FXIII-A on fibrinolysis in a plasma environment [150]. Several fibrinolytic inhibitors can be cross-linked into the forming clot including α_2_AP [151], thrombin-activatable fibrinolysis inhibitor (TAFI) [44] and plasminogen activator inhibitor-2 (PAI-2) [152]. The principal inhibitor of plasmin, α_2_AP, is synthesized in the liver and secreted as methionine (Met1-α_2_AP). In plasma, the N-terminal 12 amino acid residues are rapidly cleaved by an antiplasmin cleaving enzyme (APCE) [153] transforming it to Asn1-α_2_AP [154]. Only the Asn1-α_2_-PI isoform is a good substrate for FXIIIa [155]. Our laboratory has shown that the dominant antifibrinolytic action of FXIIIa is mediated exclusively by cross-linking α_2_AP to fibrin [142] with negligible contribution of the other inhibitors. Rijken and colleagues subsequently reported that compaction or retraction of fibrin clots reveals the strong antifibrinolytic effect of FXIII-A [156]. The authors also confirm our observations that cross-linking of α_2_AP is required for the antifibrinolytic effect of FXIII to be visualised rather than by fibrin–fibrin cross-links [156]. It is likely that the dominant effect of FXIIIa on fibrinolysis is mediated via α_2_AP with cross-linking of fibres playing a minor contribution.

Platelet FXIII-A was previously shown to stabilise clots, by inducing the formation of high molecular weight γ-dimer and α-polymer [157,158,159,160,161] and cross-linking α_2_AP to fibrin [157,160]. The conundrum is that FXIII-A is not found within the secretome of platelets. We have shown that strong agonist stimulation of platelets induces translocation of FXIII-A from the cytoplasm to the platelet membrane where it is actively retained and can participate in extracellular cross-linking reactions [64]. The intensity of FXIII-A staining on the surface of activated platelets increases as a function of time, particularly in those platelets directly associated with collagen fibres. Our work clearly highlights a role for externalised platelet FXIII-A in stabilizing thrombi via cross-linking of α_2_AP to fibrin [64]. Intriguingly, plasma FXIII-A, but not platelet FXIII-A, aids the retention of red blood cells in clots via fibrin α-chain cross-linking which has a direct impact on the overall size of clots [90,162,163]. The relative contribution of plasma FXIIIA_2_B_2_ versus platelet-derived FXIII-A to thrombus stability requires clarification, but it is unlikely to be uniform throughout the thrombus, with the balance tipping toward FXIII-A in platelet-rich areas of the haemostatic plug, where solute transport of the large plasma FXIIIA_2_B_2_ tetramer is low. Together with the fact that levels of FXIII-A are around 150-fold higher in the platelet cytoplasm, this advocates a role for these anucleate cells in thrombus stabilisation in certain environments.

### 5.2. FXIII in Wound Healing

Normal wound healing occurs in response to a haemostatic challenge or necrosis with infection [164] and involves formation of a provisional matrix which is the basis for invasion of cells involved in tissue regeneration. Impaired wound healing occurs in around 15–30% of FXIII-deficient patients [122,165,166]. Elegant studies with FXIII-A-deficient mice show prolonged healing of excisional wounds and delayed tissue repair which could be rectified by infusion of FXIII concentrate in the mice [167]. A rat model of experimental colitis showed a significant improvement of existing and established lesion severity following intravenous infusion of recombinant FXIII-A [168]. These lines of evidence highlight the crucial function of this transglutaminase in wound healing and remodelling.

**Figure 4:**
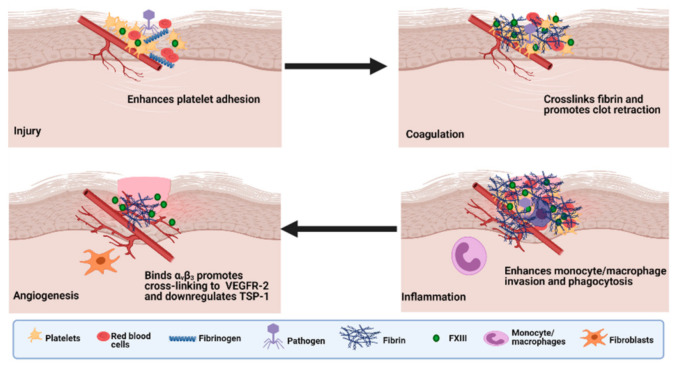
The roles of Factor XIII in wound healing. During injury, FXIII-A enhances platelet adhesion to the injured endothelium through an integrin-dependent mechanism. Activated FXIII (FXIIIa) mediates the cross-linking of fibrin and promotes clot retraction. FXIII-A enhances invasion of monocytes/macrophages and phagocytosis of cell debris and pathogens. FXIII-A promotes crosslinking of extracellular matrix (ECM) and reduces vascular permeability. FXIIIa binds α_v_β_3_ and promotes cross-linking to vascular endothelial growth factor receptor-2 (VEGFR-2) and downregulates thrombospondin-1 (TSP-1). Adapted from the work in [133].

The contribution of FXIII-A to wound healing and tissue repair is pleiotropic (as reviewed in [137,169]) and commences with its crucial function in the haemostatic cascade in terms of platelet adhesion to the sub-endothelium, which is mediated by the integrins αIIbβ3 and αvβ_3_ on the platelet surface, but occurs in a transglutaminase independent manner [170] (Figure 4). It subsequently stabilises the forming fibrin matrix and consolidates the clot by participating in clot retraction. Consequently, FXIII-A reduces vascular permeability at the wound and traps invading pathogens by crosslinking them to the provisional matrix. Finally, it promotes repair by supporting cellular invasion and stimulates angiogenic signalling. The many substrates of FXIIIa include adhesive, extracellular matrix proteins, such as fibronectin, vitronectin, thrombospondin, collagen and von Willebrand factor, which are cross-linked into the clot [169] and enhance cell migration and attachment [171]. Indeed, early studies showed that cross-linking of fibronectin into the fibrin clot via FXIII-A enhances fibroblast adherence and migration [172]. FXIII-A binds to endothelial cells via the integrin α_V_β_3_ enhancing platelet adhesion at the site of injury [173]. Binding occurs via a tripeptide motif Leu-Asp-Val in FXIII-A independent of transglutaminase activity thereby permitting ongoing cross-linking of proteins involved in repair to occur in a localised manner [170].

Macrophages are key to tissue repair and play essential roles in removal of cell debris, invading organisms, neutrophils and other apoptotic cells from the injured site. FXIII-A participates in phagocytic processes including Fcγ and complement-induced uptake of sensitized erythrocytes and complement-coated yeast particles [174]. However, it is clear that macrophages play a complex and intricate role in tissue repair supplying many chemokines, matrix metalloproteinases and other inflammatory mediators that drive the cellular response to injury [175]. Indeed, activated FXIII-A has been shown to generate complement C5-derived monocyte chemotactic factor [176]. A protein that is indistinguishable from ribosomal protein S19 has been shown to be converted to an active form via crosslinking of FXIII-A to the surface of activated platelets [177]. These chemotactic factors formed via cross-linking dependent processes may function to actively recruit monocytes and inflammatory macrophages to the site of injury.

FXIII-A reduces endothelial permeability in transglutaminase dependent manner in in vitro monolayers and saline-perfused rat hearts [178]. Similarly, a reduction in vascular permeability was noted in an in vivo guinea pig model of antiserum-induced vascular damage [179]. In patients undergoing cardiac surgery, infusion of FXIII concentrate reduces vascular leakage [180,181]. Together these findings indicate that FXIII-A protects endothelial barrier function most likely via cross-linking of adhesive proteins to the site of injury.

Angiogenesis is an important part of tissue repair and wound healing [137]. FXIII-A exerts a direct proangiogenic effect on endothelial cells in vitro, promoting migration and proliferation while constraining apoptosis [182]. FXIII-A transamidase activity is a requirement to illicit these proangiogenic effects which were associated with downregulation of the antiangiogenic factor thrombospondin-1. The process is multifaceted with binding of FXIII-A to endothelial cells inducing complex formation between vascular endothelial growth factor (VEGF) and the integrin α_V_β_3_ (vitronectin receptor). Association of FXIII-A with α_V_β_3_ results in partial cross-linking between the β_3_ subunit and the VEGFR-2 [183]. This promotes a cascade of events including tyrosine phosphorylation and activation of VEGFR-2 accompanied by upregulation of intracellular signalling molecules, such as *c-Jun* and *Egr-1* and a subsequent downregulation of thrombospondin-1 [183]. These actions are entirely dependent on the transamidase activity of FXIII-A. In addition, the interplay between the extracellular matrix (ECM) and integrins is important for the migration of reparative cells into the wound bed and the transmission of intracellular signals caused by extracellular changes [184]. These observations provide a clear link between FXIII-A of plasma and cellular origin in the wound healing and tissue repair process, although evidently this is an intricate process of which there are still several missing links that require further insight and elucidation. There is potential for application of FXIII-A to promote wound healing which may prove beneficial in some context, such as during inflammatory conditions, post-operative bleeding and trauma.

## 6. FXIII-A Replacement Therapy and Utility as a Drug Target

The management of FXIII deficiency includes regular prophylaxis [185] with replacement therapy to increase the amount of FXIII in plasma by administering cryoprecipitate, fresh frozen plasma (FFP) [186], heat inactivated concentrate or recombinant FXIII-A_2_ (rFXIII-A_2_) therapy [187]. FXIII has relatively long half-life (~9 days), making it suitable for routine prophylactic treatment given around every 4 weeks [188]. The use of FFP and cryoprecipitate carries a risk of bloodborne virus transmission, so FXIII concentrates are viewed a safer alternative as they undergo rigorous screening and viral inactivation [189].

Prophylactic treatment aims to have a trough level greater than 5% FXIII obtained with doses of 35 to 40 U/kg [190]. A target level of 8–9% of plasma FXIII was thought to be sufficient to maintain normal haemostasis [117,191,192]. However, to maintain thrombus stability against fibrinolysis, around 50% normal plasma concentrations are required [150]. Fibrogammin P^®^ is a purified heat treated FXIII concentrate used to treat congenital FXIII deficiency and is well tolerated but requires regular intravenous doses. Fibrogammin P^®^ was approved for use in Europe since the early 1990s and subsequently in USA under the name Corifact™. The plasma-derived product is approved for use with both FXIII-A subunit and the rarer FXIII-B subunit deficiency. Preoperative prophylaxis with the concentrate is effective in preventing postoperative bleeding [193]. The recombinant FXIII-A (rFXIII-A) subunit product, termed Tretten, is produced in yeast and therefore benefits from not involving any human or mammalian products in its manufacture. Upon infusion it complexes with the excess of endogenous FXIII-B in plasma generating a heterotetramer with a similar half-life to native FXIII [194]. Long-term safety and efficacy of rFXIII-A were evaluated in the Mentor™2 extension trial and demonstrated a low incidence of bleeding, no reports of development of non-neutralising or neutralising antibodies and that pre-surgery prophylaxis was effective [195].

Congenital deficiency of FXIII-A in pregnancy is generally managed with more regular, low dose (10 IU/kg) prophylaxis [196], ideally aiming for higher than 10–12 IU/d with above 30% plasma FXIII being ideal during labour [123,197]. A bolus of 1000 IU is recommended prior to onset of labour to prevent postpartum haemorrhage [123]. Recently, rFXIII-A_2_ has been shown to be effective at successfully facilitating a healthy pregnancy [197]. Prompt prophylaxis in neonates with FXIII concentrate is effective at both low (10–26 IU/kg) and high (60/80 IU/kg) doses with those on the higher dose having less bleeding episodes and no incidences of thrombotic events [196].

Acquired FXIII deficiency can result from a decreased production, increased consumption or, more rarely, due to the development of neutralising or non-neutralising autoantibodies [198]. Autoantibodies can be categorised depending how they interfere with FXIII: type Aa both block formation of the tetramer and steal FXIII-A, while Ab blocks the active transglutaminase and B rapidly clears the FXIII–antibody complex [199]. Treatment therefore requires immunosuppression, in addition to FXIII concentrate. Treatments are varied, but the steroid prednisolone is commonly prescribed combined with an immunosuppressant such as cyclophosphamide or the anti-CD20 monoclonal antibody Rituximab [198,200]. Other treatments include plasmapheresis and targeting fibrinolysis with tranexamic acid and epsilon aminocaproic acid [198,200].

FXIII replacement therapy has recently assimilated interest in the field of trauma. Trauma-induced coagulopathy (TIC) is associated with a dramatic decline in fibrinogen over other coagulation factors [201,202,203]. Consequently, there is a concomitant decrease in FXIIIA_2_B_2_ which circulates in complex with fibrinogen [6]. Early replacement of fibrinogen is associated with improved outcomes in TIC [204,205,206]. In vitro models of TIC have suggested that there is benefit to replacing not only fibrinogen, but also FXIII to enhance clot stability [207,208]. Patient studies and clinical trials of available sources of FXIII, including concentrate, fresh frozen plasma and cryoprecipitate, will be necessary to extrapolate on its potential benefit in this setting. Similarly, low levels of FXIII-A have been implicated in intraoperative unexplained bleeding episodes [209] indicating the potential of FXIII-A supplementation for perioperative bleeding. Bleeding volume after cardiac surgery shows a strong correlation with FXIII-A activity [210,211]. Pre-operative supplementation with FXIII-A has been shown to be effective in improve clot stiffness and is associated with reduced bleeding [212].

Current anticoagulant therapies are targeted indirectly or directly to downregulate thrombin production, thereby attenuating platelet activation and fibrin formation. However, the common anticoagulants, which are vitamin K-mediated or target-specific coagulation factors, generally FXa or thrombin, are associated with bleeding complications. FXIII-A has been considered a promising therapeutic strategy, as it is downstream of thrombin, and therefore permits clot formation but promotes instability, thereby enhancing susceptibility to clearance. In a canine coronary thrombosis model, pretreatment with the FXIIIa inhibitor L-722,151 (2-[l-acetonylthio]-5-methylthiazolo[2,3-b] 1,3,4-thiadiazolium, effectively enhanced tPA-induced reperfusion and reduced thrombus mass but no benefit was observed administering the inhibitor post-thrombus formation [213]. The authors suggested L-722,151 could be a pharmacological tool and as prototype for the development of future therapeutic FXIIIa inhibitors [213]. Tridegin, a small peptide inhibitor purified from the salivary gland extract of the giant Amazon leech *Haementeria ghilianii*, inhibits both plasma and platelet FXIIIa without interfering with the enzymatic activity of thrombin or Factor Xa [214]. Analogues of this peptidic inhibitor offer insight into the mechanism of action and have potential as lead structures for development [215]. The novel inhibitors with a *cis*-bisamido epoxides pharmacore were shown to have an improved potency compared to a natural product inhibitor, cerulenin, although still lacked selectivity for FXIII over transglutaminase 2 [216]. ZED3197 has a Michael acceptor warhead which irreversibly blocks the active site cysteine and has recently been shown to restore blood flow in an in vivo rabbit model of venous stasis without affecting clotting time [217]. An alternative strategy to the peptidic inhibitors is siRNA targeting of FXIII-B which causes a depletion of plasma FXIII-A, without altering platelet FXIII-A and has been shown to enhance fibrinolysis [218]. Clearly, the crucial role that FXIII-A plays in haemostasis spotlights this enzyme as a potential target for antithrombotic strategies. However, caution must be applied considering the bleeding phenotype and wound healing complications associated with congenital FXIII-A deficiency even with the mild deficiency of FXIII-B and acquired FXIII-A deficiency.

## 7. Discussion and Future Perspectives

FXIII-A is crucial to normal physiology as indicated by the bleeding diathesis of deficient patients, which can range from relatively mild to devastatingly fatal in terms of the intracranial haemorrhage associated with congenital FXIII-A deficiency in the absence of FXIII supplementation. This is amalgamated with the vital role of FXIII-A in wound healing, that was perhaps difficult to tease out considering the overbearing impact on the haemostatic cascade. The key roles of FXIII-A in normal biological processes are of course embodied within corresponding pathophysiological processes. These have not been discussed herein, as are expertly discussed in another review in this series on FXIII-A in diseases [219]. A growing body of literature now propels the function of cellular pools of FXIII-A into the limelight. These cells are known to externalise FXIII-A and therefore are capable of delivering extracellular functions of this complex transglutaminase. It is clear we have many unanswered questions to tackle, starting with the mechanisms by which FXIII-A escapes the cells despite its lack of signal sequence. This knowledge will impart us with a clearer understanding of the processes that drive externalisation and the relative contribution of cellular sources in different settings and biological processes. Of interest is the influence of different circulatory pools of FXIII-A to haemostasis in varying locales of the vasculature. The role of FXIII-A in modulating wound healing and tissue repair is now unequivocal, but the mechanisms underpinning this are still very much in their infancy. It appears that the field of FXIII-A is ripe for development particularly with the wealth of potential therapeutic options exploding into the market.
